# *Penicillium digitatum*, First Clinical Report in Chile: Fungal Co-Infection in COVID-19 Patient

**DOI:** 10.3390/jof8090961

**Published:** 2022-09-14

**Authors:** Isabel Iturrieta-González, Annesi Giacaman, Patricio Godoy-Martínez, Fernando Vega, Marcela Sepúlveda, Cledir Santos, Valentina Toledo, Gonzalo Rivera, Leandro Ortega, Andrés San Martín, Vitalia Bahamondes, Felipe Collao, Raúl Sánchez, Flery Fonseca-Salamanca

**Affiliations:** 1 Department of Preclinic Sciences, Medicine Faculty, Laboratory of Molecular Immunoparasitology, Center of Excellence in Translational Medicine-Scientific and Technological Nucleus (CEMT-BIOREN), Universidad de La Frontera, Temuco 4810296, Chile; 2 Jeffrey Modell Center of Diagnosis and Research in Primary Immunodeficiencies, Center of Excellence in Translational Medicine, Faculty of Medicine, Universidad de La Frontera, Temuco 4810296, Chile; 3 Instituto de Microbiología Clínica, Facultad de Medicina, Universidad Austral de Chile, Valdivia 5110566, Chile; 4 Unidad de Paciente Crítico, Hospital Dr. Hernán Henríquez Aravena, Temuco 4781151, Chile; 5 Department of Chemical Science and Natural Resources, Universidad de La Frontera, Temuco 4811230, Chile; 6 Subdepartamento Laboratorio Clínico, Hospital Dr. Hernán Henríquez Aravena, Temuco 4781151, Chile; 7 Department of Preclinic Sciences, Medicine Faculty, Center of Excellence in Translational Medicine-Scientific and Technological Bioresource Nucleus (CEMT-BIOREN), Universidad de La Frontera, Temuco 4810296, Chile

**Keywords:** *Penicillium digitatum*, COVID-19, pulmonar infection, pneumonia

## Abstract

*Penicillium digitatum* is one of the most important phytopathogens. It causes deterioration and rotting of citrus fruits, generating significant economic losses worldwide. As a human pathogen, it is extremely rare. We present a case of pulmonary co-infection in a patient diagnosed with pneumonia due to SARS-CoV-2. A 20-year-old female patient, primigravid, 36 weeks of gestation, without comorbidities, and diagnosed with severe pneumonia due to the SARS-CoV-2, showed rapid lung deterioration for which their pregnancy was interrupted by surgery. The patient was hospitalized in the Intensive Care Unit (ICU), connected to mechanical ventilation and receiving corticosteroids and antibiotics. The diagnosis of pulmonary fungal infection was made through bronchoalveolar lavage (BAL) culture, and the species identification was performed by sequencing of β-tubulin. Phylogenetic analysis with related species was performed for the confirmation of species identification. Antifungal susceptibility tests were performed for itraconazole (4 µg/mL), voriconazole (2 µg/mL), and amphotericin B (2 µg/mL). The patient was successfully treated with itraconazole. This is the second worldwide report of pulmonary infection by *P. digitatum* and the first in Chile. Although it is a fungus that rarely infects humans, it could represent an emerging opportunistic fungal pathogen, with associated risk factors that should be considered in the differential diagnosis of *Penicillium* species isolated from infections in humans.

## 1. Introduction

*Penicillium digitatum* is a mesophilic fungus and one of the most devasting agents of deterioration and rotting of citrus fruits [1,2]. Together with *Penicillium italicum*, it causes about 90% of losses worldwide, mainly affecting the post-harvest stages. However, it can generate deleterious effects in all phases of production, from the cultivation of the fruits to collection, packaging, storage, transport, and market, or even after being acquired by the consumer [2]. Due to the production of ethylene, indole alkaloids, and the secretion of enzymes that soften the adjacent fruits, the infection in fruits spreads rapidly, generating complete rot in about 4–5 days, spreading easily through contact [3,4]. It acts as a necrotrophic organism generating infection through previous damage to the surface of the fruit and is recognized as an agent capable of producing a potential mycotoxin called citrinin, which is associated with nephrotoxic, embryotoxic, teratogenic, and carcinogenic effects in humans and animals [5,6,7]. 

Infections caused by *P. digitatum* in humans are extremely rare. Currently, there is only one clinical report developed in 2013 in Japan that corresponded to fatal pneumonia [8]. This study describes the second clinical report of *P. digitatum* worldwide and the first in Chile, in a patient who developed pneumonia associated with COVID-19 that was successfully treated with itraconazole.

### Case Presentation

**Figure 1:**
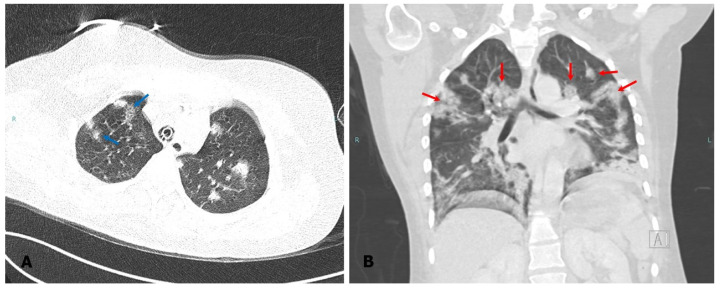
Computed tomography of the chest. The image taken prior to the diagnosis of fungal infection shows consolidation of ground glass opacities ((**A**), blue arrow) and bilateral nodular opacities in the upper lobes ((**B**), red arrow).

A 20-year-old woman from the rural area of La Araucanía region of Chile, primigravid, 36 weeks of gestation, without comorbidities, was admitted to a health center for presenting, in the prior 5 days, commitment of the general state, headache, myalgia, dry cough, progressive dyspnea, and anosmia with 10 days of evolution. On physical examination, she was found to be tachypneic, tachycardic, and saturating 88%; as the bilateral crepitus was auscultated, a chest radiograph was performed that showed bilateral interstitial compromise with basal predominance. According to clinical suspicion, SARS-CoV-2 virus detection tests were carried out, which were positive, so she was diagnosed with severe COVID-19 pneumonia. Due to the significant pulmonary compromise and thus need for oxygen therapy, and due to her advanced pregnancy condition, she was transferred to Dr. Hernán Henríquez Aravena Hospital (Temuco, Chile), where she underwent an emergency cesarean section and started antimicrobial treatment with ceftriaxone 2 g/iv/day, dexamethasone 6 mg/iv/day, enoxaparin 40 mg/sc/day. She was admitted to the ICU, where she was connected to a mechanical ventilator. Chest computed tomography (CT) on admission showed bilateral ground glass opacities and condensing opacities, consistent with organizing pneumonia. On the 10th day after surgery, she was successfully extubated and maintained with oxygen support through a High-Flow Nasal Cannula (HFNC) (50 L/min). However, she remained under surveillance, since she presented a dimer D elevation of 7.70 µg/mL, leukocytosis with 18,890 cells/µL, C-reactive protein (CRP) of 8.8 mg/L, and bilateral wheezing. Due to a worsening of her clinical condition with purulent secretions associated with poor respiratory mechanics, tachycardia, and hypertension, with a fever of 38 °C, she was reconnected to mechanical ventilation 2 days after being extubated and a new CT was performed, which showed consolidation of ground glass opacities and the appearance of new bilateral nodular opacities in the upper lobes; pulmonary embolism (PE) was ruled out (Figure 1). On the 13th day after admission, BAL was taken for a complete pathogen study that included bacterial culture, fungal culture, FilmArray (26 pathogens), galactomannan, and molecular detection of *Pneumocystis jirovecii*; peripheral blood and central venous catheter (CVC) blood cultures were also taken, and empiric therapy was started with vancomycin 2 g/iv/day for 4 days, meropenem 1 g every 8 h/iv for 7 days, and dexamethasone for 1 day. Bacterial culture was negative, molecular detection of *P. jirovecii* negative, galactomannan 0.15, Gram stain without germs and FilmArray (26 pathogens) negative. The patient persisted with fevers and pulmonary secretions, so on the 20th day after admission, a chest CT without contrast was performed, where multiple bilateral ground-glass opacities, predominantly at the bases and without pleural effusion or pneumothorax, were observed. Fungal growth was detected in fungal culture, identified by micromorphology as *Penicillium* sp. Therefore, treatment with itraconazole 400 mg for 10 days was started, followed by 200 mg/day for 28 days. After starting antifungal treatment, the patient began to evolve favorably, remaining stable, afebrile, and with decreasing inflammatory parameters. After completing 8 days with itraconazole, it was decided to disconnect her from mechanical ventilation, and the procedure was well-tolerated and uneventful. Finally, after 16 days of antifungal treatment and with good clinical and laboratory evolution, it was decided to continue antifungal treatment on an outpatient basis, and the patient was discharged from the hospital.

## 2. Materials and Methods

### 2.1. Fungal Culture and Phenotypic Identification

The fungal culture of the BAL sample was performed on potato dextrose agar (PDA) (Biokar Diagnostics, France) in duplicate, incubating at 25 °C in darkness. The micromorphological study was carried out through the microscopic observation of preparations made with Lactophenol and with cotton blue Lactophenol solutions. The images were obtained with the TissueFAXS I PLUS Cytometer TissueGnostics Axio Observer 7 Carl Zeiss GmbH (TissueGnostics GmbH, Vienna, Austria). Images were obtained in the fields of view at 40× and 63× magnifications.

### 2.2. Molecular Identification and Phylogenetic Analysis

Species identification was performed through sequencing of the phylogenetic marker β-tubulin (*BenA*) according to the recommendations of Visagie et al. [9]. For this, the fungal genomic DNA was extracted using the commercial kit Mini kit QIAamp DNA (Qiagen), according to the manufacturer’s instructions. PCR for *BenA* amplification was performed using the primer pairs T10/bt2b following previously described protocols [10], and the PCR product was purified and stored at −20 °C until sequencing. The sequences were obtained using the same primer pairs at Austral-omics of the Universidad Austral de Chile using the ABI Prism 310 automated sequencer. Sequence editing was done with SeqMan v. 7.0.0 (DNAStar Lasergene, Madison, WI, USA), and the consensus sequence obtained was submitted for comparison in the National Center for Biotechnology Information (NCBI) database using the BLASTn tool. Subsequently, the phylogenetic analysis of the β-tubulin marker was performed with inclusion of the sequences of the strain CEMT 2 together with sequences of seven ex-type strains of species representatives of the series *Clavigera, Sclerotigena, Italica, Penicillium,* and *Digitata.* The alignment of the locus was performed in MEGA software (Molecular Evolutionary Genetics Analysis) v. 6.0 [11], through Clustal W algorithm [12] and refined with MUSCLE [13] or manually if necessary. The phylogenetic analysis was performed using the maximum-likelihood method (ML) under the same software. The best nucleotide substitution model for the ML was Kimura 2-parameter with gamma distribution (K2 + G). Bootstrap values ≥ 70% were considered significant. Sequences from *Aspergillus fumigatus* CBS 133.61 and *Aspergillus clavatus* CBS 513.65 were used as outgroup (Table 1).

### 2.3. In Vitro Antifungals Susceptibility Tests 

The broth microdilution method was performed according to the guidelines of the CLSI document M38-A2 [14]. Minimum inhibitory concentration (MIC) values were determined for amphotericin B (Sigma-Aldrich, St. Louis, MO, USA), voriconazole (Sigma-Aldrich), and itraconazole (Sigma-Aldrich). Fungal inoculum suspensions were prepared in sterile distilled water from 5-day-old fungal culture on PDA at 28 °C. The suspension was filtered through sterile gauze. Cell counts were determined with a hemocytometer and the inoculum size was adjusted to around 10^5^ conidia/mL. Stock solutions of antifungals were prepared in 100% dimethyl sulfoxide (DMSO) to 100-fold the final concentration needed and further diluted in RPMI 1640 pH 7.0 (Sigma-Aldrich Co., St. Louis, MO, USA) to obtain the ×2 drug concentration. Dilutions of the fungicides were prepared and dispensed into 96-well microdilution plates. Each microdilution well containing 100 μL of the appropriate antifungal solution (2× final concentration) was inoculated with 100 μL of the conidial inoculum suspension, yielding final antifungal concentrations of 0.06, 0.12, 0.24, 0.5, 1, 2, 4, 8, 16, and 32 μg/mL. The growth control wells contained 100 μL of the inoculum suspension and 100 μL of RPMI medium. Sterility control wells contained 200 μL of RPMI medium. For determination of MIC, microdilution plates were incubated at 27 °C and visually examined using a stereoscopic magnifier (SZ61TR, OLYMPUS) from 0 up to 48 h from the time of inoculation. MIC was defined as the lowest concentration of each antifungal agent that causes a specified reduction in visible growth of the fungal strain on the broth dilution susceptibility test. The reference strains *Fusarium oxysporum* (ATCC 36031) and *Fusarium keratoplasticum* (ATCC 48112) were used as quality control for the test.

### 2.4. Ethics Statement

The present study has the approval of the Ethics Committee of Servicio de Salud Araucanía Sur (protocol code N° 26 approved on 25 January 2022). Informed consent was obtained from the patient involved in the study. 

## 3. Results

### 3.1. Phenotypic Study and Preliminary Identification 

**Figure 2:**
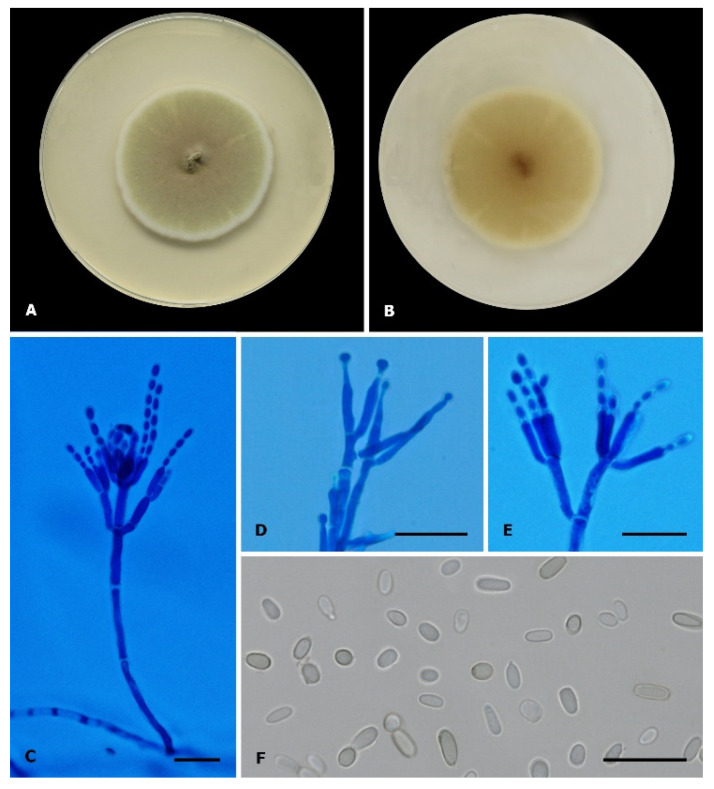
*Penicillium digitatum* (CEMT-2). (**A**,**B**) Colony on PDA, in front and reverse respectively, incubated at 25 °C for 7 days. (**C**–**E**) Conidiophores, metulae, phialides, and conidia (Cotton blue Lactophenol mounting solution). (**F**) Conidia (Lactophenol mounting solution). Scale bars: 20 µm.

On the PDA culture medium, fungal development was observed after 7 days of incubation at 25 °C with the development of velvety colonies with a greenish hue (Figure 2A). The microscopic characterization of the isolate (CEMT 2) was performed via observation of specimen mounted in Lactophenol and cotton blue Lactophenol. The observation of septate hyaline hyphae, from which conidiophores, metulae, phialides and oval conidia-forming chains originated, allowed us to identify the isolate as *Penicillium* sp. (Figure 2C–F)

### 3.2. Molecular Identification and Phylogenetic Analysis 

**Figure 3:**
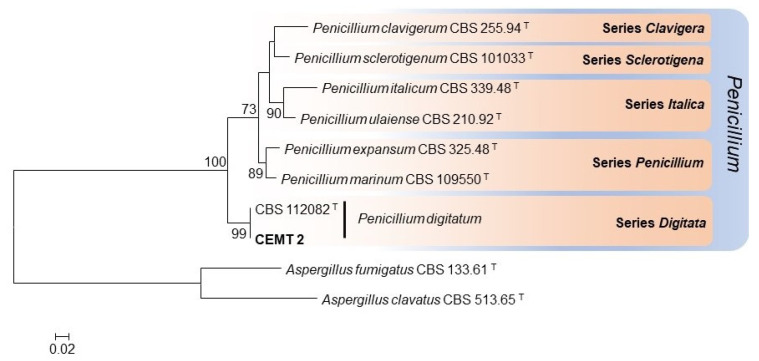
Maximum-likelihood phylogenetic tree constructed with β-tubulin sequences from 7 representative species of the series *Clavigera, Sclerotigena, Italica, Penicillium*, and *Digitata*. Bootstrap values ≥ 70% are indicated in the nodes. The fungal strain of the present study is indicated in bold. Branch lengths are proportional to phylogenetic distance. T: Ex-type species.

The molecular identification confirmed the preliminary identification and defined the strain as *Penicillium digitatum*, which presented 100% identity with several reference strain sequences of that species and 99.75% identity with the sequence of the type-strain (*P. digitatum* CBS 112082). The sequence data generated in the present study were deposited in GenBank (Table 1). The phylogenetic analysis carried out with the type-strain of the series *Digitata* (series that includes the identified species) and other phylogenetically related series (*Penicillium*, *Italica*, *Sclerotigena* and *Clavigera*) confirmed the identification, showing the formation of a well-supported clade (bootstrap of 99%) made up of the strain of the present study (CEMT 2) and the type-strain of *P. digitatum* (CBS 112082). The phylogenetic relationships between our isolate and other related species are shown in Figure 3.

### 3.3. Results of In Vitro Antifungal Susceptibility Test

The susceptibility profiles obtained showed an in vitro activity of the three antifungal drugs studied, with low MIC values ranging between 2 and 4 µg/mL, amphotericin B and voriconazole being the ones that showed better activity. MIC values for amphotericin B, voriconazole, and itraconazole are shown in Table 2.

## 4. Discussion

Invasive fungal infections (IFI) have shown a considerable increase in recent decades, constituting an emerging problem worldwide. Although they mainly affect patients with risk factors such as hematologic malignancies, solid organ and bone marrow transplants, acquired immunodeficiency syndrome (AIDS), corticosteroid treatment, extensive use of antibiotics, CVC, chemotherapy, or mechanical ventilation, among several others [15,16], serious fungal infections have also been described in immunocompetent individuals [17,18,19,20,21,22].

Aspergillosis undoubtedly constitutes the most frequent IFI, not only in patients with the aforementioned risk factors, but also in patients with pulmonary infection by SARS-CoV-2. In this last group, cases of co-infection have been reported worldwide, even establishing the concept of COVID-19-associated pulmonary aspergillosis (CAPA) [23]. Although COVID-19 patients are not immunocompromised patients as such, they present a series of risk factors that make them susceptible to the development of infections by other agents, whether bacterial or fungal. Among them, local pulmonary hypoxia, a product of the viral infection itself, and damage to the respiratory epithelium, mechanical ventilation, prolonged hospitalizations, and therapy with broad-spectrum antibiotics and immunosuppressive drugs, particularly systemic corticosteroids, are of great relevance, since together they contribute to the settlement and germination of external agents, facilitating colonization and invasion [24,25,26].

Infections by non-*Aspergillus* filamentous fungi are considerably less frequent and also constitute a challenge not only from the point of view of their treatment, due to the high levels of resistance that some species present, but also due to the difficulty in their correct identification, a product of the increasing number of cryptic species described in various fungal genera [27]. Regarding the genus *Penicillium*, until a few years ago, the species *Penicillium marneffei* was described as the most frequent in clinical infections in humans. However, this species is currently classified within the genus *Talaromyces* (*T. marneffei*), so the clinical reports caused by other *Penicillium* species are very rare. Lyratzopoulos et al. [28] conducted a review of 31 cases of invasive fungal infection by *Penicillium* species, among which they described *P. chrysogenum, P. decumbens P. janthinellum, P. lilacinum, P. purporogenum, P. citrinum,* and *P. brevicompactum* as the majority of them, isolated from invasive pulmonary infection [28].

*Penicillium digitatum* is a fungus with worldwide distribution, widely recognized as a phytopathogen in citrus fruits, with clinical isolates being extremely rare [2]. There has only been one previous report made in Japan, in which this species was described as an agent of pneumonia with a fatal outcome, in an elderly patient who was suffering from pulmonary emphysema and malnutrition [8]. Although the patient in the current case reported not having the habit of consuming citrus fruits very frequently, she could have acquired it from any other external source, even in her own home, precisely because it is an environmental fungus. This is why it is very difficult to establish the source of contagion in this type of organism with certainty. Although the patient was immunocompetent and without underlying diseases, she was suffering from a pulmonary infection due to SARS-CoV-2, requiring the administration of oxygen therapy, which conditioned the interruption of her pregnancy through surgery, hospitalization in ICU, installation of CVC, connection to mechanical ventilation, and treatment with corticosteroids and antibiotics—all of these being risk factors that facilitate the development of IFI. Co-infection between SARS-CoV-2 and fungal species has been reported in various studies worldwide [24,29,30,31,32,33]. The genus *Aspergillus* appears as the most frequent agent [23,34,35,36]; however, the present study highlights the pathogenic potential of other emerging fungal species in patients diagnosed with COVID-19. 

Regarding the diagnostic tests recommended in COVID-19 patients for the diagnosis of CAPA, it is important to highlight the detection of galactomannan antigen, which constitutes an important component of the cell wall of *Aspergillus* spp., secreted in vivo during the growth of the fungus [37,38]. Therefore, its detection has high sensitivity and specificity for the diagnosis of this fungal infection; however, its usefulness in invasive infections by other genera is limited. Based on the consensus criteria established by ECMM/ISHAM for the diagnosis of CAPA, a patient with pulmonary infiltrates associated with an optical density index (ODI) value in BAL ≥ 1.0 could be diagnosed with a possible CAPA [39]. In this case, since the ODI in BAL was 0.15, it was possible to rule out CAPA, but not an invasive pulmonary infection caused by another type of fungus.

Unfortunately, in the report by Oshikata et al. [8], antifungal susceptibility tests were not carried out; however, they described *P. digitatum* as a species resistant to antifungals, an assertion based only on the clinical evolution of the patient due to a poor response despite the administration of itraconazole, micafungin, voriconazole, and amphotericin B. Our results disagree with what was described by Oshikata et al. [8], since the MIC values found were in general lower, 2 µg/mL for amphotericin B and voriconazole, or slightly higher, 4 µg/mL for itraconazole. Although amphotericin B is described as a good therapeutic option in cases of invasive fungal infection by *Penicillium* non-*marneffei* species [28], in this report and unlike that described by Oshikata et al. [8], only itraconazole was administered with good therapeutic success, showing high levels of susceptibility not only in vitro, but also in vivo. In any case, it is essential to analyze a larger number of isolates of clinical and environmental origin in order to establish an antifungal susceptibility profile in this species and determine if there is intra-species variability. 

Although this species still has a low clinical frequency, it clearly implies a greater burden on the health system, generating higher costs, prolonged hospitalizations, and greater work absenteeism. In the present study, we describe the first clinical isolation of *P. digitatum* in Chile, being the second clinical report worldwide, postulating it as an emerging opportunistic fungal pathogen that should be considered in the differential diagnosis of *Penicillium* spp. isolated from infectious diseases in humans. 

## Figures and Tables

**Table 1:** *Penicillium* species included in the phylogenetic study, their respective origin, and GenBank accession number.

Species	Strain	Substrate	Locality	β-tubulin GenBank Accession Numbers
*Aspergillus fumigatus*	CBS 133.61 ^T^	Chicken, lung	USA	KF314730
*A. clavatus*	CBS 513.65 ^T^	Unknown	Unknown	EU076340
*Penicillium clavigerum*	CBS 255.94 ^T^	Man	Canada	AY674427
*P. expansum*	CBS 325.48 ^T^	*Malus sylvestris*, fruit	USA	AY674400
*P. digitatum*	CBS 112082 ^T^	*Citrus limon*	Italy	KJ834447
	**CEMT 2**	**Human Bronchoalveolar Lavage**	**Chile**	**OP046418**
*P. italicum*	CBS 339.48 ^T^	*Citrus* sp., fruit	USA	AY674398
*P. marinum*	CBS 109550 ^T^	Sandy soil	Japan	AY674392
*P. sclerotigenum*	CBS 101033 ^T^	*Dioscorea batatas*, rotting tuber	Japan	AY674393
*P. ulaiense*	CBS 210.92 ^T^	skin of decaying orange	Taiwan	AY674408

Note: Newly generated sequences in this study are indicated in bold. Abbreviations: CBS, culture collection of the Westerdijk Fungal Biodiversity Institute, Utrecht, the Netherlands; CEMT, Centro de Excelencia en Medicina Traslacional, Universidad de La Frontera, Temuco, Chile; ^T^, ex-type strain.

**Table 2:** Results of in vitro antifungal susceptibility test of the strain *Penicillium digitatum* CEMT-2.

Fungal Strain	Antifungal	MIC (µg/mL)
*Penicillium digitatum* CEMT-2	Amphotericin B	2
	Voriconazole	2
	Itraconazole	4
*Fusarium oxysporum* ATCC 36031	Amphotericin B	2
	Voriconazole	16
	Itraconazole	>32
*Fusarium keratoplasticum* ATCC 48112	Amphotericin B	4
	Voriconazole	32
	Itraconazole	>32

Abbreviations: ATCC, American Type Culture Collection; CEMT, Centro de Excelencia en Medicina Traslacional, Universidad de La Frontera, Temuco, Chile.
